# Templated Synthesis and Assembly of Two-, Three- and Six-Patch Silica Nanoparticles with a Controlled Patch-to-Particle Size Ratio

**DOI:** 10.3390/molecules26164736

**Published:** 2021-08-05

**Authors:** Bin Liu, Stéphanie Exiga, Etienne Duguet, Serge Ravaine

**Affiliations:** 1 Univ. Bordeaux, CNRS, CRPP, UMR 5031, 33600 Pessac, France; bin.liu-bin@u-bordeaux.fr (B.L.); stephanie.exiga@crpp.cnrs.fr (S.E.); 2 Univ. Bordeaux, CNRS, Bordeaux INP, ICMCB, UMR 5026, 33600 Pessac, France

**Keywords:** patchy nanoparticles, silica, patch size, assembly

## Abstract

We report a fabrication route of silica nanoparticles with two, three or six patches with an easily tunable patch-to-particle size ratio. The synthetic pathway includes two main stages: the synthesis of silica/polystyrene multipod-like templates and the selective growth of their silica core through an iterative approach. Electron microscopy of the dimpled nanoparticles obtained after dissolution of the polystyrene nodules of the multipod-like nanoparticles provides evidence of the conformational growth of the silica core. Thanks to the presence of some polymer chains, which remained grafted at the bottom of the dimples after the dissolution of the PS nodules, the solvent-induced assembly of the patchy nanoparticles is performed. Chains, hexagonal suprastructures and cubic lattices are obtained from the assembly of two-, three- and six-patch silica nanoparticles, respectively. Our study can guide future work in both patchy nanoparticle synthesis and self-assembly. It also opens new routes towards the fabrication of specific classes of one-, two- and three-dimensional colloidal lattices, including complex tilings.

## 1. Introduction

In the last decades, tremendous efforts have been made towards the development of synthetic strategies for hybrid organic–inorganic (nano)materials, on the one hand [1,2,3], and for anisotropic particles with well-controlled size, shape and composition, on the other hand [4,5,6,7,8,9,10,11]. Most of the latter has been aimed at producing building blocks for the bottom-up construction of novel functional materials and devices with more complex and predictable architectures, which could have applications in photonics [12] and optics [13]. One of the most effective strategies towards achieving high degrees of synthetic control consists of using a pre-existing template. For instance, emulsion droplets have been often used as template to synthesize hollow particles: silica capsules have been prepared from Pickering emulsions [14] or in oil-in-water emulsions [15], and iron oxide hollow spheres have been synthesized in glycerol-in-water quasi-emulsion [16]. Colloidal particles can also serve as a template to synthesize hollow or hybrid structures. As an example, Xia et al. demonstrated the conformational coating of positively charged polystyrene (PS) beads with silica [17]. They also reported that when negatively charged PS beads were employed as templates, the silica coating was not homogeneous and silica colloids with non-spherical morphologies were produced. Titania hollow spheres were obtained through deposition of titania on the surface of silica particles by hydrolysis of titanium butoxide and condensation with the assistance of hydroxypropyl cellulose, which served as a surfactant [18]. Chen et al. demonstrated the formation of Janus particles by modifying the surface of gold nanoparticles with poly-acrylic acid and 4-mercaptophenylacetic acid acting as competing surfactants [19]. Janus particles can be considered as one-patch colloids if one of the two hemispheres is sticky. More generally, multi-patch particles are very interesting for their ability to assemble with their fellows by controlling the number of particles in contact and the orientation of the bonds according to the well-known valence principle for atoms in covalent compounds [20]. We reported that multivalent silica particles with well-controlled size and shape could be obtained from multipod-like silica/PS nanoparticles acting as templates through the slow addition of an ethanol solution of tetraethoxysilane (TEOS), which induced the conformational growth of their silica core [21]. More recently, we showed that the continuous addition of larger quantities of TEOS did not allow us to extend the re-growth of the silica core of tetrapod-like particles. Indeed, TEOS molecules migrate inside the PS nodules when their concentration is too high, leading to the formation of excrescences on the surface of the nodules [22]. To avoid the undesired formation of these silica bumps, we developed an alternative iterative route based on successive additions of a small amount of TEOS and centrifugation/redispersion cycles to eliminate the excess of unreacted TEOS.

Here, we report that we successfully extended this strategy to bi-, tri- and hexapod-like templates, allowing us to fabricate silica particles with 2, 3 or 6 PS patches, respectively. By carefully analyzing the particles by electron microscopy, we show that we could precisely control the patch-to-particle size ratio by varying the added amount of TEOS. The specific dissolution of PS gave rise to silica nanoparticles with a precise number of dimples, which combine both enthalpic and entropic patchiness thanks to the presence of grafted PS chains at the bottom of the cavities [23]. Taking advantage of the stickiness of the latter in a good/bad solvent mixture, we successfully assembled the patchy nanoparticles into chains, hexagonal suprastructures and simple cubic lattices.

## 2. Results

**Scheme 1:**
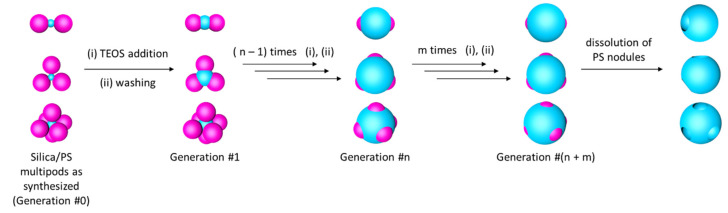
Synthetic route for the preparation of particles with two-, three- and six-PS patches of controlled size from the corresponding silica/PS multipods and the subsequent formation of particles with concave patches through dissolution of the PS lobes.

Scheme 1 shows the multi-step approach developed to synthesize patchy silica particles with controlled patch size from PS/silica multipods. It consists of growing the silica core of the silica/PS multipods used as templates through successive additions of a small amount of TEOS interspersed with centrifugation/redispersion cycles in order to eliminate the excess of unreacted TEOS molecules and the formation of silica protuberances at the surface of the PS lobes, which occurs when TEOS is added at once [22].

**Figure 1:**
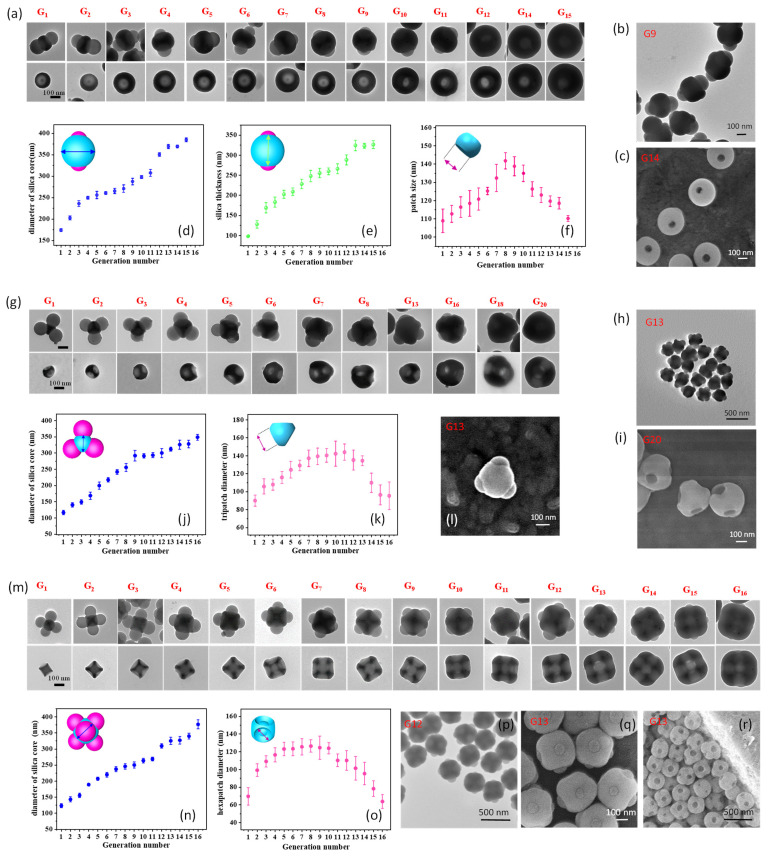
(**a**) TEM images of the bipod-like particles obtained after 1 to 15 iterative silica growth steps (corresponding to generations #1 to #15). The bottom row shows images of the corresponding two-patch particles obtained after dissolution of the PS nodules. All images are at the same scale; (**b**) Low magnification TEM image of the bipod-like particles after 9 iterative silica growth steps; (**c**) Scanning electron microscopy (SEM) image of the two-patch particles of generation #14; (**d**,**e**) Evolution of the diameter and the thickness of the silica core as a function of the generation number; (**f**) Evolution of the patch size as a function of the generation number; (**g**) TEM images of the tripod-like particles obtained after 1 to 20 iterative silica growth steps. The bottom row shows images of the corresponding three-patch particles obtained after dissolution of the PS nodules. All images are at the same scale; (**h**) Low magnification TEM image of the tripod-like particles after 13 iterative silica growth steps; (**i**) SEM image of the three-patch particles of generation #20; (**j**) Evolution of the diameter of the silica core as a function of the generation number; (**k**) Evolution of the patch size as a function of the generation number; (**l**) SEM image of a tripod-like particle of generation # 13; (**m**) TEM images of the hexapod-like particles obtained after 1 to 16 iterative silica growth steps. The bottom row shows images of the corresponding six-patch particles obtained after dissolution of the PS nodules. All images are at the same scale; (**n**) Evolution of the diameter of the silica core as a function of the generation number; (**o**) Evolution of the patch size as a function of the generation number; (**p**) Low magnification TEM image of the hexapod-like particles after 12 iterative silica growth steps; (**q**) SEM image of the hexapod-like particles of generation #13; (**r**) SEM image of the six-patch particles of generation #13.

Transmission electron microscopy (TEM) images in Figure 1a,g,m show the morphology of the bi-, tri- and hexapod-like silica/PS nanoparticles after up to twenty iterative silica growth steps. In all cases, one can see that the silica core grew by strictly following the shape of the PS nodules and no silica bump formed on the surface of these, confirming the relevance of the chosen multi-step synthetic approach. The diameter of the silica core increased homogeneously as a function of the successive additions of TEOS, as shown in Figure 1d,e,j,n. Morphological yields were determined by statistical analysis of low-magnification images (Figure 1b,h,p,q) over about 100 nanoparticles. For generations #15, yields were equal to 81%, 78%, and 68% for bi-, tri- and hexapods, respectively. Considering the uncertainties linked to the statistical analyses, these values turn out to be very close to the initial morphological purity of the batches of multipods (see Table 1), which demonstrates that the regrowth stage of the silica core does not contribute to the morphological polydispersity of the batches.

The subsequent dissolution of the PS nodules of the multipod-like nanoparticles gave rise to silica particles with a fixed number of concave patches in a well-controlled geometry, i.e., linear, triangular or cubic (Scheme 1 and Figure 1a,c,g,i,m,r). The values of the concave patch size were calculated for each generation from the TEM images. Figure 1f,k,o show that the patch size first started to increase to reach a maximum and then decreased while the number of TEOS additions increased. This result confirms the conformational growth of the silica core, i.e., that the silica core encapsulated the PS nodules while it grew, as its diameter must increase until it reaches the equator of the PS lobes and decreases when this point is overpassed.

**Figure 2:**
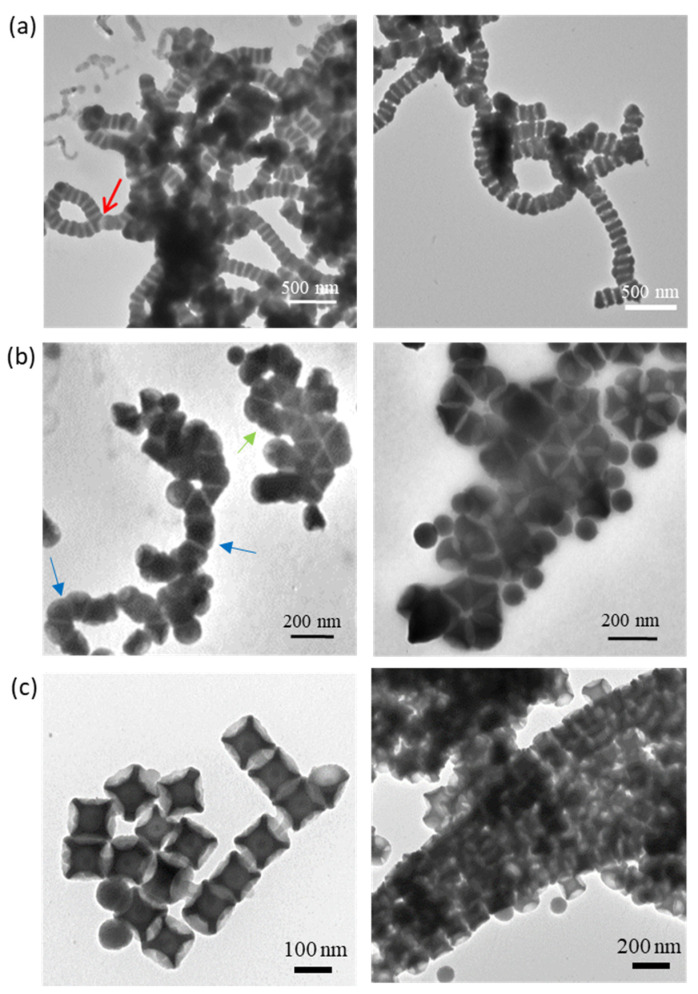
(**a**) TEM images of colloidal chains obtained after incubating two-patch silica nanoparticles for 19 h into a 7/3 (*v/v*) THF/salty water mixture. (**b**) TEM images of chains and hexagonal superstructures obtained after incubating three-patch silica nanoparticles for 16 (left) and 40 (right) hours into a 9/1 (*v/v*) THF/water mixture. (**c**) TEM images of assemblies resulting from the incubation of six-patch nanoparticles for 16 (left) and 40 (right) hours into a 9/1 (*v/v*) THF/water mixture.

A careful examination of the TEM images of the silica nanoparticles after dissolution of the PS nodules ((Figure 1a,g,m, bottom rows) reveals the presence of a dark spot in the middle of the dimples. Thanks to energy-dispersive X-ray spectroscopy (EDX) analyses reported in a previous report, we have shown that it corresponds to the presence a few PS chains with number-average mass higher than 500,000 g mol^−1^, which are covalently grafted onto the initial silica seed surface [23]. We took benefit of the presence of these PS “residues” that are expected to become sticky in a good/bad solvent mixture to induce the self-assembly of the patchy silica nanoparticles. Salty water was firstly added into a two-patch silica nanoparticles (Generation #1) dispersion in THF, in order to reduce both the electrostatic repulsions between nanoparticles due to negatively charged silanolate groups at their surface (the ζ potential of the patchy silica nanoparticles was −42 mV at pH 5.7) and solvent quality for the PS chains. The later ones therefore formed physical bonds to minimize the free surface energy of the system [24,25] and chains resulting from the side-by-side assembly of the two-patch nanoparticles were obtained after 19 h of incubation (Figure 2a). Some chains grew up to a few microns and contained up to ~50 building units. Branching of the chains could also be observed (red arrow) and was attributed to the presence of a small fraction (~15%) of three-patch nanoparticles as byproducts in the batch of two-patch nanoparticles, which act as branching points. Following the same recipe, we successfully obtained chains by incubating two-patch nanoparticles of generation #2, but it was not possible to assemble silica nanoparticles of higher generation numbers. We assume that it is because the PS chains located at the bottom of the dimples are not long enough to interact when the silica core became too thick. This result tends to indicate that enthalpic interactions between polymer patches have a predominant role in the nanoparticle assembly and that shape-induced entropic interactions [26,27] are not sufficient to induce assembly under our experimental conditions. Short chains were also obtained by adding water into a three-patch silica nanoparticles (Generation #1) dispersion in THF and incubating for 16 h (Figure 2b, blue arrows). These 1D assemblies resulted in the formation of edge-to-edge connections between the patchy silica nanoparticles. More interestingly, hexagonal entities consisting of densely packed patchy nanoparticles forming edge-to-edge and point-to-point connections were also observed (Figure 2b, green arrows). At longer incubation times, these hexagons assembled into larger two-dimensional suprastructures (Figure 2b, right) similar to those previously observed during the self-assembly of gold nanotriangles [28,29] or beveled gold triangular nanoprisms [30], which can be considered as the early stages of the formation of a planar honeycomb lattice. Unfortunately, the presence of impurities (two- and four-patch nanoparticles) strongly hinders the formation of larger ordered patterns. As shown in Figure 2c, six-patch silica nanoparticles (Generation #1) assembled into one-dimensional assemblies which form right angles after 14 h of incubation into a 9/1 (*v/v*) THF/water mixture. The spacing between some patchy nanoparticles, probably caused by drying during the preparation of the TEM grid, allowed us to clearly visualize the interparticular attachment of the PS chains, which were made sticky by reducing the solvent quality. At longer times, compact arrays exhibiting a simple cubic structure could be observed (Figure 2c, right) as expected [31,32], even if the presence of impurities such as nanoparticles with four or five patches (see Table 1) strongly inhibits the extension of the ordered lattice. For both three- and six-patch nanoparticles, no assembly was observed by incubation of nanoparticles issued of generation #3 and beyond, which confirms the primordial role of the enthalpic patches located at the bottom of the cavities of the silica nanoparticles. When the concentration of patchy particles was divided by two (all the more so by ten), only a few small chains (for 2- or 6-patch NPs), and hexagonal entities (for 3-patch NPs) were observed, as the probability for the NPs to collide with each other is much lower. When the concentration of NPs was multiplied by 4, similar assemblies were observed in all cases.

## 3. Materials and Methods

### 3.1. Materials

We used styrene (99%), sodium persulfate (99%), Symperonic^®^ NP30, sodium dodecylsulfate (SDS, >90%), tetraethoxysilane (TEOS, 99%), l-arginine (98.5%) and ammonium hydroxide (28–30% in water), as we received them from Sigma Aldrich (Saint-Quentin-Fallavier, France). We purchased absolute ethanol from VWR International S.A.S (Fontenay-sous-Bois, France) and methacryloxymethyltriethoxysilane (MMS, 98%) from abcr GmbH (Karlsruhe, Germany) and used them as received. We systematically used ultrapure water with a resistivity of 18.2 MΩ·cm at 25 °C obtained from a Milli-Q system (Merck Millipore, Molsheim, France).

### 3.2. Synthesis of the Silica/PS Multipods

We prepared batches of bipods, tripods and hexapods consisting of a central silica core surrounded by two, three and six PS satellite nodules, respectively, by seeded-growth emulsion polymerization of styrene (Table 1), according to a procedure we published previously [33]. We used silica seeds previously surface-modified with MMS (0.5 molecule·nm^−2^), a 95%/5% *w/w* surfactant mixture (3 g·L^−1^) of Symperonic^®^ NP30 and SDS, and sodium persulfate as initiator (0.5 % *w/w* relative to monomer). The polymerization was performed at 70 °C for 6 h.

### 3.3. Controlled Growth of the Silica Core

Totals of 9.1 mL of absolute ethanol, 0.7 mL of ammonia and 0.2 mL of the dispersion of silica-polystyrene multipods were introduced into a 25 mL flask and the mixture was homogenized using a magnetic bar. Then, 200 µL of TEOS was added all at once after 5 min. The reaction was left under stirring at 20 °C for 15 min. The reaction medium was poured into a 50 mL Falcon tube containing 15 mL of absolute ethanol. After 2 cycles of centrifugation (12,000× *g*/5 min) and redispersion in absolute ethanol, the nanoparticles were finally redispersed in 10 mL of a previously prepared hydro-alcoholic solution (absolute ethanol/ammonia/water volume ratio: 91%/7%/2%). This protocol was renewed to obtain the next generation.

### 3.4. Dissolution of the PS Nodules

For dissolving the PS nodules of the multipods, 100 µL of the particle dispersion was subjected to two centrifugation/redispersion cycles in THF (8000× *g*/10 min; 200 μL). The final concentration of patchy NPs in THF was adjusted to 3.6∙× 10^14^ part/L and the solution was kept at 4 °C.

### 3.5. Assembly of the Patchy Particles

Incubation of the particles was carried out in 15 mL tubes under rolling motion at 60 rpm at room temperature. For reducing the quality of the THF solvent, a calculated volume of water for three- and six-patch nanoparticles or salty water (20 mM NaCl aqueous solution) for two-patch nanoparticles was added drop-by-drop under stirring to reach a fraction of 10 vol.% (for three-patch and six-patch nanoparticles) or 30 vol.% (for two-patch nanoparticles) and a total volume of 2 mL. It took about 20 s to add 100 μL. Assembled structures were monitored by collecting at various incubation times 50 µL samples and direct deposition on TEM grids for liquid evaporation before observation.

### 3.6. Characterization Techniques

Transmission electron microscopy (TEM) and scanning electron microscopy (SEM) experiments were performed using a Hitachi H600 microscope operating at an acceleration voltage of 75 kV and a Hitachi S4500 microscope with an accelerating voltage of 5 kV, respectively. We prepared the samples by depositing one drop of the colloidal dispersion on conventional carbon-coated copper grids. We let the liquid evaporate in the open air at room temperature and placed the grids in a box away from dust. Before SEM analysis, samples deposited on a 300-mesh carbon coated copper grid were coated with nm-thick layer of gold and palladium (10%) with a SC7620 mini sputter coater.

The ζ potential value of a two-patch silica nanoparticles aqueous dispersion (10^15^ nanoparticles·L^−1^, pH~5.7) was measured using the Malvern Zetasizer 3000 HS setup (Malvern Instruments, Malvern, UK). The dielectric constant of water was set to 80.4 and the Smoluchowsky constant f(ka) was 1.5.

## 4. Conclusions

In conclusion, the conformational enlargement of the silica core of bi-, tri- and hexapods allowed us to synthesize silica nanoparticles with two, three or six circular PS patches of controlled size, respectively. The incubation of the patchy nanoparticles in a mixture of a good and a bad solvent for PS made the patches sticky, which induced the assembly of the patchy silica nanoparticles in the form of chains, hexagonal suprastructures and simple cubic lattice depending on the number of patches at their surface. Even if homogeneity and purity of the patchy nanoparticles are major challenges, which must be taken into account, extending such a strategy to mixtures of particles with different numbers of patches could lead to the fabrication of new nonconventional ordered assemblies such as quasicrystals and thus contribute to enriching diversity in self-assembly structures. Another prospect concerns the direct imaging of patchy nanoparticle self-assembly in liquid, which could help to better understand the interparticle interaction governing dynamic assembly process. Liquid-phase TEM technique can be a powerful tool to achieve this challenging perspective.

## Figures and Tables

**Table 1:** Characteristics of the multipod-like precursors.

Precursors	Diameter of Silica Core (nm)	Diameter of PS Nodules (nm)	Morphology Purity ^1^
Bipods	55 ± 2	160 ± 6	79% bipods, 15% tripods, 6% monopods
Tripods	55 ± 2	145 ± 6	77% tripods, 9% bipods, 14% tetrapods
Hexapods	85 ± 2	130 ± 5	81% hexapods, 15% pentapods, 4% tetrapods

^1^ determined by statistical analysis of TEM images.

## Data Availability

The data presented in this study are available on request from the corresponding authors.

## Sample Availability

Samples of the patchy silica nanoparticles are available from the authors.
